# Intra-Rater and Test–Retest Reliability of Kinovea for the Kinematic Analysis of Squatting in Healthy Active Women

**DOI:** 10.3390/s26123749

**Published:** 2026-06-12

**Authors:** Concepción Vicente-Loren, María Orosia Lucha-López, Sofía Monti-Ballano, Sergio Hijazo-Larrosa, Lucía Vicente-Pina, Loreto Ferrández-Laliena, José Miguel Tricás-Moreno, César Hidalgo-García

**Affiliations:** 1 O2 Centro Wellness Boutique Gym, Don Ramón de la Cruz, 28001 Madrid, Spain; 2 Unidad de Investigación en Fisioterapia, Departamento de Fisiatría y Enfermería, Universidad de Zaragoza, Domingo Miral, 50009 Zaragoza, Spain; smonti@unizar.es (S.M.-B.); shijazo@unizar.es (S.H.-L.); l.vicente@unizar.es (L.V.-P.); lferrandez@unizar.es (L.F.-L.); jmtricas@unizar.es (J.M.T.-M.); hidalgo@unizar.es (C.H.-G.)

**Keywords:** Kinovea, reliability, squat, kinematics, minimum detectable change, 2D video analysis, women

## Abstract

The squat is a critical component of numerous rehabilitation and functional assessment protocols, playing a significant role in enhancing athletic performance and activities of daily living. Although some of the characteristics gathered during the squat need additional confirmation, Kinovea provides a free two-dimensional squat motion analysis tool that is simple to use in clinical practice. This analytical, cross-sectional reliability study aimed to evaluate the intra-rater and test–retest reliability (with a 20 min interval between performances) of loaded squat kinematics in a sample of women using Kinovea. Twenty women performed a loaded back squat; intra-rater reliability was assessed by re-analyzing the same video one week apart, and test–retest reliability was assessed across two performances separated by 20 min. The results showed good to excellent intra-rater reliability (ICC: 0.75–0.99; SEM: 0.16 cm to 5.14°; MDC: 0.44 cm to 14.24°), and moderate to excellent test–retest reliability (ICC: 0.64–0.98; SEM: 0.36 cm to 14.29°; MDC: 0.99 cm to 39.61°). Variables tracked in the sagittal plane showed high precision. Conversely, the head angle and knee angle in the frontal plane exhibited greater variability, reflected by higher SEM and MDC values. In conclusion, Kinovea is a reliable and accessible tool for clinical kinematic assessment of the squat, particularly in the sagittal plane parameters. However, due to the elevated measurement error observed in head angles and frontal-plane knee dynamics, the integration of 3D motion capture is recommended over 2D digital protocols for these variables.

## 1. Introduction

The squat is regarded as one of the most efficacious workouts for enhancing athletic performance, as it necessitates the coordination of substantial muscle groups engaged in activities such as jumping, running, or weightlifting [1]. Beyond the athletic context, squat is a fundamental functional action for activities of daily living, such as sitting, lowering oneself to the floor or lifting weights from a low height [2,3]. Indeed, it has been suggested as an effective tool for identifying lower limb biomechanical deficiencies [2]. Due to its dual role as a functional movement and a strength exercise, it is frequently incorporated into knee, hip or ankle rehabilitation programs [4,5,6,7].

According to biomechanical definitions, the squat is a multi-joint exercise in a closed kinetic chain that demands concurrent coordination of the hip, knee, and ankle joints. It entails extending the lower limbs to lower the center of gravity toward the base of support while preserving the vertical alignment of both the trunk and the spine [1,4,5]. The ability of the central nervous system to precisely and effectively coordinate and adjust the activation of various muscle groups and joint segments during a workout is referred to as a motor control [6,7,8]. However, the NSCA observes that athletes often exhibit insufficient technique or control, thereby elevating the risk of injury or re-injury, particularly when subjected to substantial external loads [9]. For instance, the knee joint presents one of the highest injury rates during squats [10]. Similarly, excessive lumbar flexion during the squat can be highly injurious to the intervertebral discs, promoting disc degeneration and low back pain [11,12].

Differentiating between male and female biomechanics is crucial when analyzing human movement. Significant physical differences exist, such as the shorter and broader pelvis in women, which increases the Q-angle and predisposes them to greater knee valgus [13,14,15,16]. Additionally, hormonal changes increase ligamentous laxity and range of motion, which frequently leads to decreased joint stability requiring additional effort from the neuromuscular system to stabilize the segments [14,17,18]. Additionally, the different phases of the menstrual cycle can alter stabilization capacity [19,20]. Finally, research demonstrates variations in muscle recruitment patterns: women tend to exhibit quadriceps dominance, whereas men show a greater reliance on the gluteal and hamstrings muscles [16,21].

The literature highlights all these factors among the limitations of previous studies due to sample heterogeneity; consequently, there is growing recognition of scenarios where research conducted exclusively with women is both ethically and methodologically justified [22]. Despite this evidence, it is only recently that there has been an increase in the number of publications focusing exclusively on the biomechanics of the squat in women [23,24,25,26,27].

Although three-dimensional (3D) motion analysis remains the gold standard, its high cost and complexity often limit its feasibility in daily clinical practice [28]. The need for accessible, reliable and low-cost biomechanical analysis tools in clinical practice motivates the evaluation of open-source software such as Kinovea (version 2023.1.1 2D video analysis).

For this clinical study to be comprehensive, it necessitates not only accessible technology but also a meticulous evaluation of body segments that, despite their functional significance, have historically been marginalized. It is important to highlight that, despite the squat’s definition encompassing the preservation of spinal neutrality, there is a paucity of research that particularly examines head position. Donnelly et al. [29] examined the impact of gaze direction (upwards, forwards or downwards) on squat kinematics, although failed to quantify segmental head displacement. Therefore, the incorporation of this variable in the current study yields innovative and pertinent data to the existing literature.

Consequently, the aim of this study is to evaluate the intra-examiner reliability and test–retest reliability of back-squat, using the Kinovea software in recreationally trained female lifters. We hypothesized that Kinovea would yield ICC ≥ 0.75 (good reliability) for sagittal-plane joint angles, with poorer reliability for frontal-plane and cervical variables.

## 2. Materials and Methods

### 2.1. Study Type and Methods

This is an analytical observational, cross-sectional reliability study conducted at a sport center.

The study protocol was approved by the Research Ethics Committee of the Community of Aragon (code PI23/444) and in accordance with the Declaration of Helsinki.

### 2.2. Participants

The inclusion criteria were set as participants aged over 18, exclusively female recreational back-squat lifters without competitive participation, members of the O2 Centro Wellness Boutique gym (Madrid).

The exclusion criteria were those who had experienced musculoskeletal injuries in the ankles, knees, hips, or spine within the preceding three months, as well as those who had undergone surgical procedures or possessed osteosynthetic or orthotic devices, including insoles. Additionally, women exhibiting discomfort symptoms or hindering the skin markers during the squat evaluation were removed.

### 2.3. Sample Size Calculation

The sample size was calculated using the method developed by Walter et al. [30] for reliability studies involving two measurements. Based on previous Kinovea reliability studies in lower-limb kinematics [31,32], this approach was used to determine the required number of subjects using the intraclass correlation coefficient (ICC). Assuming values for type I and type II errors of α = 0.05 and β = 0.20, respectively, a minimum acceptable ICC of 0.70 and an expected ICC of 0.90, it was estimated that 18.4 subjects were required. Finally, the study was conducted with 20 participants.

### 2.4. Outcome Variables and Instrumentation

A self-administered questionnaire was used to gather sociodemographic data in order to categorize the sample. These variables included age, occupation, daily sitting time, years of athletic experience, weekly training volume (hours), sport type, and whether or not mobility exercises were included in training sessions. Anthropometric data were measured using a mechanical tape measure (Seca@ 206) for height and a digital diagnostic scale (Beurer@ SR-BF788) for body weight. The Body Mass Index (BMI = weight (kg) [height (m)^2^]) was computed using this data [33].

The kinematics of the movement were recorded during two critical phases of the exercise:

- Position 1 (P1): the subjects’ position with the bar behind the neck (upper trapezius), immediately before beginning the knee flexion.
- Position 2 (P2): position of maximum knee flexion for each subject (“maximum squat”), without pain and without lifting the heels off the ground.

**Figure 1:**
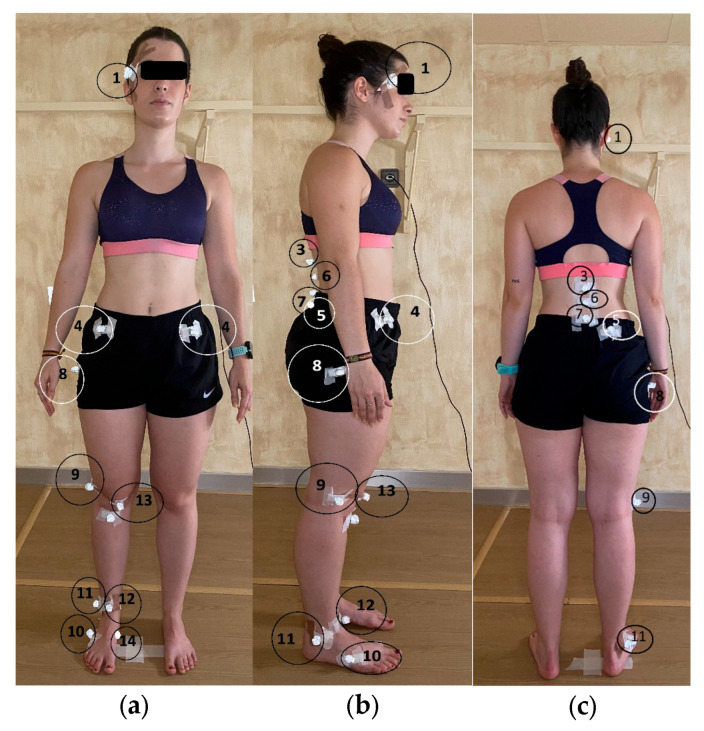
Placement of markers: (**a**) frontal plane, anterior view; (**b**) sagittal plane; (**c**) frontal plane, posterior view.

The two-dimensional (2D) motion capture software Kinovea^®^ (version 2023.1.1) (available at: https://www.kinovea.org/download/ accessed on 30 November 2023) was used for the analysis. The recording system consisted of two Logitech^®^ C920S HD Pro cameras (Logitech Europe S.A., Lausanne, Switzerland) installed on different tripods. Video files were recorded in MKV format and imported directly into Kinovea without compression to preserve image quality. Videos were captured at a sampling rate of 30 frames per second (fps). The frontal and sagittal planes could be simultaneously captured since the cameras were positioned perpendicular to one another, 90 cm above the floor, and 1.5 m from the central point of execution [34]. Both cameras were connected to a computer that managed data processing, enabling the evaluator to record both planes synchronously. For image calibration, reflective control markers (10 cm × 5 cm) were placed on the walls facing each camera at a height of 90 cm, positioned where they would not be obstructed by the participant during the squat. Spatial calibration in Kinovea was performed for each camera view by using the known dimension of these reference wall markers to establish the pixel-to-centimeter ratio within the movement plane. The software’s origin of coordinates (0.0) was default-set at the top-left corner of the video frame, with the horizontal axis parallel to the floor. The subjects stood on a cross-mark on the floor that ensured the right foot was positioned on the optical axis of the cameras. To enable analysis, 2 × 2 cm reflective markers were placed by the same examiner at the following anatomical and reference points (Figure 1):

- Lateral aspect of the temple (extension of the right orbital line) (1);
- Visible end of the squat bar (2);
- Tip of the spinous process of T12 (3);
- On clothing, over the left and right anterior superior iliac spines (ASIS) (4);
- On clothing, over the right posterior superior iliac spine (PSIS) (5);
- Tip of the spinous process of L3 (6);
- Tip of the spinous process of S1 (7);
- On the clothing at the level of the greater trochanter of the right femur (8);
- On the lateral epicondyle of the right femur (9);
- On the styloid process of the fifth metatarsal of the right foot (10);
- At the anatomical center of the lateral malleolus of the right ankle (11);
- Midpoint between the tibial and fibular malleoli of the right leg (12);
- At the anatomical center of the right patella (13);
- On the tubercle of the scaphoid bone of the right foot (14).

In addition to the markers, a barbell and weight plates were required to apply the load. The barbell was 130 cm long and weighed 1 kg. The weight plates were 5 kg and 2.5 kg. Determining the barbell load involved careful consideration. Although 1RM testing is common in the literature, it was ruled out since the participants were untrained with maximum loads. Instead, based on the protocol by List et al. [35] a standardized load of 25% of the participant’s body weight was adopted [35].

The variables recorded in the sagittal plane were:

- Head angle: this measurement was used to observe the behavior of the head and cervical spine. The angle was formed between a vertical line originating from marker 2 (the end of the squat bar viewed from a sagittal plane) and a line connecting to marker 1 (placed on the extension of the eye on the side being examined) [29]. If the angle increased compared to the initial measurement, the subject had altered their cervical position towards flexion, whereas if the angle decreased, the change in the position of the cervical spine was towards extension.
- Lumbar lordosis: this angle measured any change in the curvature of the lumbar spine during the squat. For this measurement, the angle obtained between the line of the T12-L3 markers (markers 3 and 6) and the L3-S1 line (markers 6 and 7) was calculated, with L3 (marker 6) as the vertex of the angle. If the angle of the lumbar spine increased, the subject performed a spinal flexion; whereas, if it decreased, the subject had extended the lumbar spine [36].
- Horizontal pelvic alignment from a sagittal plane: this was measured using the pelvic horizontal alignment (PHA) parameter, the angle formed between the line joining the anterior superior iliac spine (ASIS—marker 4) (vertex of the angle) and the posterior superior iliac spine (PSIS—marker 5) and a horizontal line parallel to the floor [37,38]. An increase in this value compared to the baseline measurement indicated that the pelvis had shifted towards anteversion, whilst a decrease indicated pelvic retroversion [37].
- Trunk forward tilt: this measurement was used to quantify the degree of forward trunk tilt for each subject relative to a line perpendicular to the ceiling. This was measured using marker 8, positioned on the greater trochanter of the femur, which acts as the vertex of the angle, marker 2, at the end of the squat bar, and a vertical line to the ceiling. If the angle increased compared to the initial measurement, this indicated that the subject had produced a trunk flexion [18,29]. Negative values at P1 indicate a slight posterior trunk lean relative to the vertical.
- Hip flexion relative to the ASIS: this value quantified the amount of hip flexion produced during the squat. The angle formed between a perpendicular line (angle in green in Table 1) passing through the line connecting the ASIS (marker 4) and the PSIS (marker 5) and the line passing through the greater trochanter (marker 8) and the lateral epicondyle of the femur (marker 9) (angle shown in yellow in Table 1). Each of the markers was positioned at these anatomical landmarks [39].
- Knee flexion: this value indicated the joint position of the knee during the squat in a sagittal view. The angle was measured between the extension of a line originating from marker 9, placed on the lateral epicondyle of the femur (vertex of the angle) and passing through marker 8 on the greater trochanter of the femur, and another line originating from the marker placed at the vertex and passing through marker 12 on the lateral malleolus of the ankle [29,39]. The measurement of this angle was key to establishing P2 during video analysis.
- Ankle flexion: this value indicated the angular change produced during the squat at the ankle. The measurement was taken as the angle formed between the lines connecting marker 9, located at the lateral epicondyle of the knee, and marker 12 on the lateral malleolus of the ankle, and marker 10 on the styloid process of the fifth metatarsal and marker 12 on the lateral malleolus of the ankle [18]. The smaller this angle, the greater the ankle dorsiflexion.

Variables recorded from the frontal plane:

- Medial-lateral knee deviation: the angle was measured formed by the intersection of the line between ASIS (marker 4) and marker 13 at the center of the patella (vertex of the angle) and the line between marker 13 at the center of the patella and marker 12 at the midpoint between the two malleoli of the ankle. If the value of this angle was negative, knee valgus was observed, whereas if the angle was positive, the knee had shifted towards varus [40,41].
- Horizontal alignment of the pelvis from a frontal plane: this measurement indicated whether the subject was able to maintain symmetry in the frontal plane between both hemipelvis. A line parallel to the floor was drawn from marker 4 located on the right ASIS, and from there another line connecting the left ASIS. The angle between these two lines was taken as an absolute value, regardless of whether it was positive or negative in the image, as it was not possible to distinguish whether the asymmetry originated from one hemipelvis or the other. The greater the angular value, the greater the pelvic asymmetry in the frontal plane.
- Foot pronosupination: To quantify this variable, a test known as the navicular drop was used [42]. This involves measuring the distance from marker 14 on the tubercle of the scaphoid to the ground [43]. If the distance from the marker to the ground decreased during movement, this indicated a drop in the plantar arch and, consequently, foot pronation. If there had been an increase in this distance, the foot had shifted into supination.

### 2.5. Procedures

Every measuring methodology was completed in a single session. Anthropometric and sociodemographic information was gathered from each participant before to the photography. Participants were required to wear tight-fitting shorts and a sports top, and the test was conducted barefoot to avoid the influence of footwear. Reflective markers were used once the process was explained, and the external stress on the barbell was calculated. For the sagittal plane analysis, the right lower limb was selected for all participants, based on Endo et al. [39], who found no significant differences between the two sides in ankle, knee and hip kinematics during the squat. The participant stood on the floor markings, positioning her feet shoulder-width apart. After checking her posture, five warm-up squats were performed with the loaded barbell.

The assessor simultaneously recorded both cameras using Kinovea whilst the subject performed three repetitions. Participants performed the squats at a self-selected tempo. Squat depth was not standardized to a fixed anatomical criterion; instead, participants were instructed to reach their maximum knee flexion during each repetition. The repetition with the highest knee flexion was selected for analysis as the participant’s maximum squat. The videos from the frontal and sagittal views were stored separately for subsequent analysis. Joint angles and postural variables were measured manually by the evaluator using the software’s angle tool at the exact frame corresponding to the participant’s maximum squat depth. Finally, the calculated angular data were manually extracted from the software interface and compiled into a spreadsheet.

**Figure 2:**
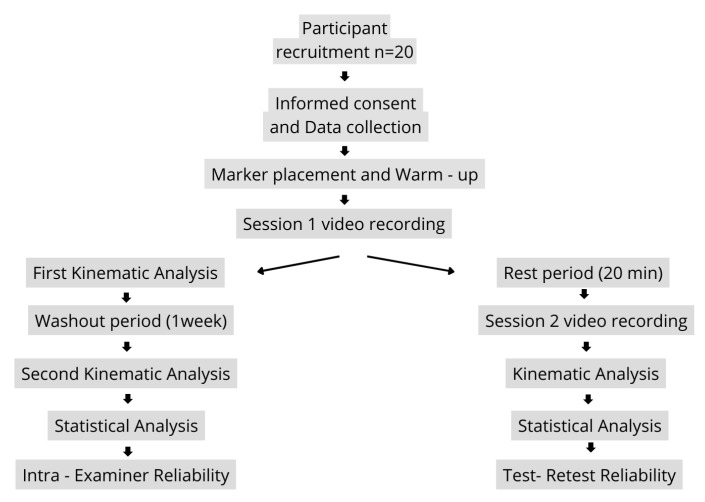
Flowchart of the study design, outlining the participant selection and the protocols for both intra-rater and inter-session reliability analyses.

In the intra-rater reliability study, the same video of a squat was analyzed at two different times, one week apart, to mitigate evaluator memory bias (Figure 2).

To determine the test–retest reliability of the measurements, two different performances by the same participant were analyzed (Figure 2). Following the first recording, a 20 min rest interval was allowed (to attempt to mitigate the participant’s movement imitation bias) before repeating the protocol (including the warm-up). During this time, the markers remained attached to the skin to ensure that any variability arose from the subject’s movement and not from the repositioning of the markers.

### 2.6. Statistical Analysis

We used IBM SPSS Statistics (v. 30) and Microsoft Excel 16 to look at the data. Measures of central tendency (mean), dispersion (standard deviation, SD), and maximum and minimum values for the quantitative variables were used in a descriptive study of the sample. A descriptive analysis of absolute frequencies was conducted for the qualitative variables.

Data normality was assessed using the Shapiro–Wilk test. While some kinematic variables showed a normal distribution (*p* > 0.05), others deviated significantly from normality (*p* < 0.001). The variables were P2 horizontal pelvic alignment from the sagittal plane, P2 medial-lateral knee deviation, P2 foot pronosupination, P2 horizontal alignment of the pelvis in the frontal plane and P2 lumbar lordosis. Despite this non-normality in specific parameters, the parametric approach was maintained because the intraclass correlation coefficient (ICC) is robust to mild violations of normality [44]. However, considering this fact, Bland–Altman plots have been included for the main variables (knee flexion, hip flexion and trunk tilt), for the variables showing violations of normality, as well.

Reliability was assessed using two complementary approaches. Relative reliability was evaluated using the intraclass correlation coefficient (ICC). Based on the guidelines by Koo and Li (2016) [45], a two-way mixed-effects, absolute agreement, single-measures model [ICC (3,1)], was selected. ICC values were interpreted as follows: ICC < 0.50 indicated poor reliability, ICC between 0.50 and 0.75 moderate, ICC between 0.75 and 0.90 good, and ICC > 0.90 excellent. On the other hand, absolute reliability was assessed by calculating the standard error of measurement (SEM) and the minimum detectable change (MDC), along with their corresponding 95% confidence intervals (95% CI).

The standard error of measurement (SEM = SD · $\sqrt{1}-ICC$) represents the random error inherent in the measurement (variability that is not real). As for the minimal detectable change (MDC = SEM · 1.96 · $\sqrt{2}$), it establishes the threshold of change required for a difference between measurements to be considered real with 95% confidence and not the result of error [31]. The standard deviation (SD) from the first measurement was consistently used as the reference variance for all SEM and MDC calculations. To further complement this analysis, Bland–Altman plots were generated specifically for parameters presenting wide MDC (head angle and frontal-plane knee). This approach was selected to visually assess the absolute agreement and to evaluate potential systematic or bias in variables where the MDC is clinically relevant for detecting true biomechanical changes between sessions.

## 3. Results

The sample consisted of 20 female participants (37.75 ± 15.47 years [range: 20–71], BMI 25.10 ± 5.09 [range: 19.94–33.79]) who met the inclusion and exclusion criteria.

Regarding the analysis of sociodemographic variables, the average daily sedentary time was 8.95 ± 3.28 h; 80% of the participants had been engaging in physical activity for more than 6 years, and 70% reported practicing more than 4 h per week. Concerning the type of physical activity performed, 75% trained with a personal trainer, 50% trained independently, and 65% regularly attended group classes at the gym. Finally, 60% did not include mobility exercises in their routines.

The Shapiro–Wilk test revealed that most kinematic variables were normally distributed, with exceptions found in five specific variables.

**Figure 3:**
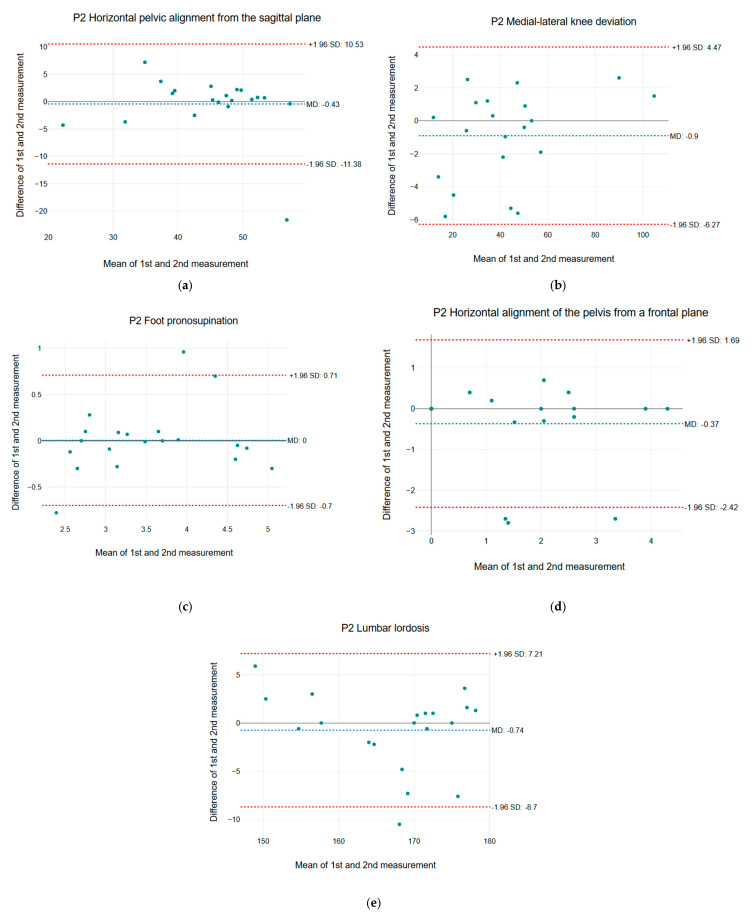
Bland–Altman plot assessing agreement between the first and second measurements. Panels (**a**–**d**) correspond to intra-rater Bland–Altman plots of non-normally distributed variables, while panel (**e**) corresponds to the test–retest analysis of lumbar lordosis. Each point represents one individual observation. The *x*-axis shows the mean of the first and second measurements for the analyzed variable, whereas the *y*-axis shows the difference between measurements, calculated as the first measurement minus the second measurement. The solid black horizontal line represents zero difference between measurements, indicating perfect agreement between the first and second measurements. The blue dashed line indicates the mean difference between measurements (MD), representing the average systematic bias. The red dashed lines indicate the 95% limits of agreement, calculated as the mean difference ±1.96 standard deviations of the differences. Bland–Altman plots demonstrating non-normally distributed kinematic variables: (**a**) horizontal pelvis alignment from sagittal plane in P2, (**b**) medial-lateral knee deviation in P2, (**c**) foot pronosupination in P2, (**d**) horizontal pelvis alignment from frontal plane in P2, and (**e**) lumbar lordosis in P2.

To assess absolute agreement and identify potential systematic bias, corresponding Bland–Altman plots were constructed (Figure 3). Specifically, panels “a, b, c and d” represent the intra-rater reliability analysis. Conversely, panel “e” illustrates the inter-session test–retest reliability.

In terms of the non-normally distributed parameters, the Bland–Altman analysis confirmed a high level of absolute consistency across all evaluated variables (Figure 3). For the intra-rater reliability block, negligible systematic biases were found for horizontal pelvic alignment in the sagittal plane (−0.43°; 95% LoA: −11.38° to 10.53°; Figure 3a), medial-lateral knee deviation (−0.90°; 95% LoA: −6.27° to 4.47°; Figure 3b), foot pronosupination (0.00 cm; 95% LoA: −0.71 cm to 0.70 cm; Figure 3c), and horizontal pelvic alignment in the frontal plane (−0.37°; 95% LoA: −2.42° to 1.69°; Figure 3d). Similarly, the test–retest analysis for lumbar lordosis (Figure 3e) reflected excellent protocol stability with a minimal systematic bias of −0.74° (95% LoA: −8.70° to 7.21°). Visual inspection of all plots revealed an optimal, homoscedastic dispersion of differences, with only an isolated outlier in panels a, c, d, and e, thereby validating the absolute reproducibility of the kinematic protocol during the maximum squat flexion phase (P2).

Regarding position and range of motion variables, a total of 40 ICCs were calculated (2 ICCs for each of the 10 variables). Of these, 20 corresponded to intra-rater reliability and the other 20 to test–retest reliability, all with a 95% confidence interval.

The intra-rater reliability analysis (Table 2) showed good to excellent consistency, with ICC values ranging from 0.75 to 0.99 (95% CI: 0.50–0.99). The trunk forward tilt (P1) and medial—lateral knee deviation (P2) demonstrated the highest reliability (ICC = 0.99). In contrast, horizontal pelvic alignment in the frontal plane (P2) showed the lowest reliability, with an ICC of 0.75 (95% CI: 0.50–0.90).

Regarding the absolute reliability and measurement precision, SEM and MDC values remained within clinically acceptable ranges for all variables. The standing pronosupination movement (P1) showed the highest measurement precision (SEM = 0.16 cm; MDC = 0.44 cm). Conversely, head angle (P1) exhibited the highest measurement error (SEM = 5.14°; MDC = 14.24°). Notably, an inspection of the reliability parameters reveals a systematic pattern across most kinematic variables: both the SEM and MDC were consistently higher at P2 compared to P1. This suggests that the biomechanical complexity and postural demands of maximal-flexion postures introduce greater digitization and marker re-identification error compared to the initial stance or shallower angles.

**Figure 4:**
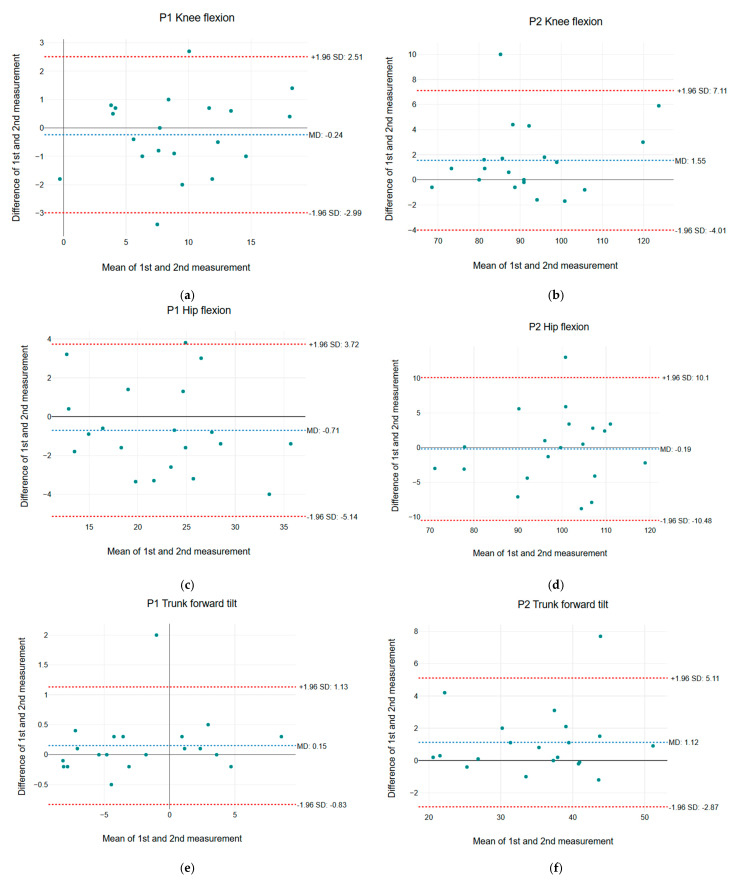
Bland–Altman plot assessing agreement between the first and second measurements. Each point represents one individual observation. The *x*-axis shows the mean of the first and second measurements for the analyzed variable, whereas the *y*-axis shows the difference between measurements, calculated as the first measurement minus the second measurement. The solid black horizontal line represents zero difference between measurements, indicating perfect agreement between the first and second measurements. The blue dashed line indicates the mean difference between measurements (MD), representing the average systematic bias. The red dashed lines indicate the 95% limits of agreement, calculated as the mean difference ± 1.96 standard deviations of the differences. Bland–Altman plots demonstrating intra-rater reliability for the primary kinematic variables: (**a**) P1 Knee flexion; (**b**) P2 Knee flexion; (**c**) P1 Hip flexion; (**d**) P2 Hip flexion; (**e**) P1 Trunk forward tilt; (**f**) P2 Trunk forward tilt.

Concerning the primary kinematic variables, the Bland–Altman analysis confirmed a strong intra-rater absolute agreement across both movement phases (Figure 4). For the initial phase (P1), negligible systematic biases and narrow error thresholds were observed for knee flexion (−0.24°; 95% LoA: −2.99° to 2.51°; Figure 4a), hip flexion (−0.71°; 95% LoA: −5.14° to 3.72°; Figure 4c), and trunk forward tilt (0.15°; 95% LoA: −0.83° to 1.13°; Figure 4e). Similarly, during the maximum flexion phase (P2), the protocol demonstrated high absolute consistency for knee flexion (1.55°; 95% LoA: −4.01° to 7.11°; Figure 4b), hip flexion (−0.19°; 95% LoA: −10.48° to 10.10 °; Figure 4d), and trunk forward tilt (1.12°; 95% LoA: −2.87° to 5.11°; Figure 4f). Visual inspection across these primary charts revealed an optimal, homoscedastic distribution of differences, with only an isolated outlier exceeding the confidence thresholds in panel d, thereby supporting the methodological precision and stability of the main joint angles.

In relation to inter-measurement test–retest reliability (Table 3), ICC values ranged from moderate to excellent with ICCs from 0.64 to 0.98 (95% CI: 0.25–0.99). Knee flexion (P2) demonstrated the highest reliability (ICC = 0.98). In contrast, horizontal pelvic alignment from a frontal plane (P2) showed the lowest reliability, with an ICC of 0.64 (95% CI: 0.25–0.80).

Regarding absolute reliability, SEM and MDC values were consistently higher than those obtained in the intra-rater assessment. The standing pronosupination movement (P1) showed the highest measurement precision (SEM = 0.36 cm; MDC = 0.99 cm), whereas the medial-lateral knee deviation (P2) exhibited the highest measurement error (SEM = 14.29°; MDC = 39.61°). In addition to this, some variables showed relatively high MDC values, including P2 hip flexion angle (MDC = 14.28°), P1 lumbar lordosis angle (MDC = 12.10°), P1 head angle (MDC = 17.65°) and P2 head angle (MDC = 26.43°).

**Figure 5:**
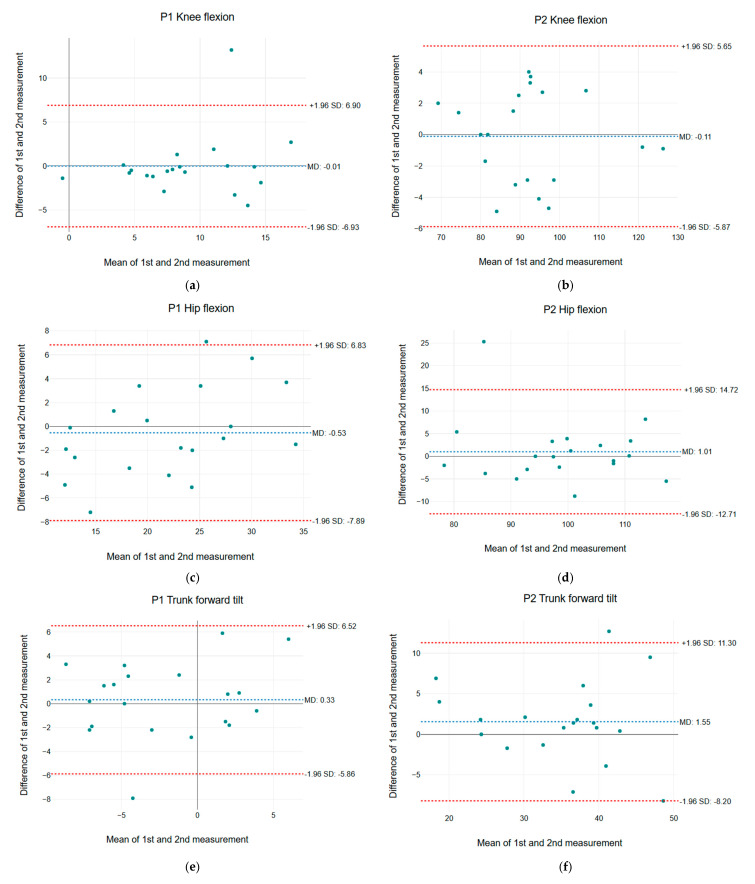
Bland–Altman plot assessing agreement between the first and second measurements. Each point represents one individual observation. The *x*-axis shows the mean of the first and second measurements for the analyzed variable, whereas the *y*-axis shows the difference between measurements, calculated as the first measurement minus the second measurement. The solid black horizontal line represents zero difference between measurements, indicating perfect agreement between the first and second measurements. The blue dashed line indicates the mean difference between measurements (MD), representing the average systematic bias. The red dashed lines indicate the 95% limits of agreement, calculated as the mean difference ± 1.96 standard deviations of the differences. Bland–Altman plots demonstrating test–retest reliability for the primary kinematic variables: (**a**) P1 Knee flexion; (**b**) P2 Knee flexion; (**c**) P1 Hip flexion; (**d**) P2 Hip flexion; (**e**) P1 Trunk forward tilt; (**f**) P2 Trunk forward tilt.

With respect to the inter-session test–retest reliability, the Bland–Altman analysis confirmed a high level of protocol stability and reproducibility for the primary joint angles across different testing days (Figure 5). For the initial phase (P1), minimal systematic biases were observed for knee flexion (−0.01°; 95% LoA: −6.93° to 6.90°; Figure 5a), hip flexion (−0.53°; 95% LoA: −7.89° to 6.83°; Figure 5c), and trunk forward tilt (0.33°; 95% LoA: −5.86° to 6.52°; Figure 5e). Similarly, during the maximum flexion phase (P2), consistency remained solid with low systematic errors for knee flexion (−0.11°; 95% LoA: −5.87° to 5.65°; Figure 5b), hip flexion (1.01°; 95% LoA: −12.71° to 14.72°; Figure 5d), and trunk forward tilt (1.55°; 95% LoA: −8.20° to 11.30°; Figure 5f). Visual inspection demonstrated a balanced and homoscedastic scatter of differences, with only an isolated outlier in panels a, d, and f transcending the confidence boundaries, thereby validating the robustness of the kinematic tracking protocol between distinct sessions.

**Figure 6:**
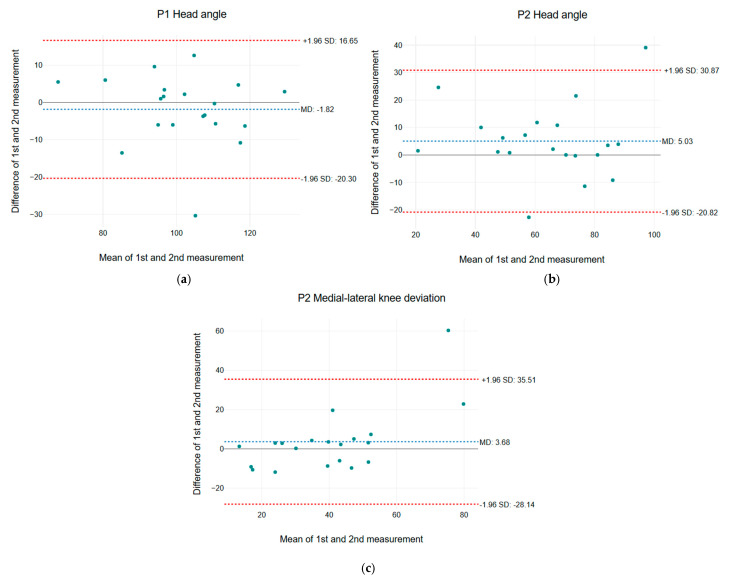
Bland–Altman plot assessing agreement between the first and second measurements. Each point represents one individual observation. The *x*-axis shows the mean of the first and second measurements for the analyzed variable, whereas the *y*-axis shows the difference between measurements, calculated as the first measurement minus the second measurement. The solid black horizontal line represents zero difference between measurements, indicating perfect agreement between the first and second measurements. The blue dashed line indicates the mean difference between measurements (MD), representing the average systematic bias. The red dashed lines indicate the 95% limits of agreement, calculated as the mean difference ± 1.96 standard deviations of the differences. Bland–Altman plots for the kinematic variables presenting the highest Minimal Detectable Change (MDC) values: (**a**) P1 Head angle; (**b**) P2 Head angle; (**c**) P2 Medial-lateral knee deviation.

In terms of the parameters presenting the highest Minimal Detectable Change (MDC) values, a dedicated Bland–Altman analysis was conducted to scrutinize their absolute precision limits (Figure 6). Concerning the cervical kinematics, the tracking of the head showed the widest error margins both during the initial phase (P1 head angle: −1.82°; 95% LoA: −20.30° to 16.65°; Figure 6a) and at the lowest point of the exercise (P2 head angle: 5.03°; 95% LoA: −20.82° to 30.87°; Figure 6b). As regards the frontal plane evaluation during maximum flexion, a higher distribution of error was also observed for the P2 medial-lateral knee deviation (3.68°; 95% LoA: −28.14° to 35.51°; Figure 6c). Despite these wider confidence intervals, visual inspection across all three plots demonstrated a balanced, homoscedastic scatter of differences, confirming that no systematic proportional bias was introduced even within the variables tracking the highest physiological variability.

## 4. Discussion

This study aimed to determine the intra-rater and test–retest reliability of the Kinovea 2D system for analyzing positional variables during the squat.

For the reliability analysis, the results demonstrated good to excellent consistency, with all analyzed kinematic variables showing ICC values between 0.75 and 0.99. These findings are highly consistent with previous literature evaluating sagittal plane kinematics with Kinovea, which uniformly reports robust reliability coefficients (ICC > 0.80) for hip, knee, and ankle angles [32], pelvic alignment [31], lumbar lordosis [46,47], and trunk tilt [48]. This systemic agreement underscores the software’s capacity to deliver highly reproducible relative tracking when standardized anatomical marker placement is maintained.

The Bland–Altman analysis (Figure 4) confirmed a strong intra-rater absolute agreement, with negligible systematic biases in the sagittal plane. The narrow limits of agreement (LoA) at P1 indicate that the standing posture provides an ultra-stable baseline for clinical tracking. Conversely, the predictable widening of error thresholds observed at P2—particularly for hip flexion—likely reflects increased tissue tension and potential displacement of skin-mounted markers at maximal joint range [49,50]. Nevertheless, visual inspection of the Bland–Altman plots did not reveal an evident heteroscedastic pattern, as the dispersion of the differences appeared relatively balanced across the range of angular values. This finding suggests that measurement variability was not clearly dependent on the participant’s angular range, supporting the potential viability of the protocol for objective clinical assessment.

When analyzing absolute reliability, SEM and MDC values obtained for horizontal pelvic alignment from a sagittal plane at P1 (SEM = 1.10°; MDC = 3.05°) were only slightly higher than those reported by Vicente–Pina [31] in bipedal position (SEM = 0.9°; MDC = 2.6°). This difference may be attributed to the additional proprioceptive and motor control demands imposed by the external load (bar) used in our protocol, which could introduce minimal variability in the initial pelvic positioning. Similar findings have been reported in studies using different measurement tools. For example, Prushansky, in a study of pelvic kinematics using digital inclinometry, reported an ICC of 0.90 and an SEM of 2° [51]. A similar pattern has been observed with portable inertial measurement units (IMUs) compared against optoelectronic systems, showing ICCs of 0.85–0.94 and SEMs between 2.7 and 8.9° in the assessment of pelvic orientation during functional activities [52,53,54].

Finally, regarding the head angle, significantly higher MDC values (14.24° at P1 and 13.21° at P2) were observed compared to the other kinematic variables. This variability may be attributed to the high degree of freedom of the head segment and the influence of gaze during exercise performance [55,56]. Furthermore, positioning the head near the edges of the camera’s field of view may have increased errors due to parallax or spherical lens distortion compared to the segments located closer to the center of the frame (e.g., pelvis and knee) [48,57]. Additionally, the observed between-session difference of >3° in the head angle at P1 warrants methodological consideration. Since the underlying physical movement was identical across both analysis sessions, this variation reflects pure operator-dependent digitization and marker re-identification error rather than kinematic variability. Nevertheless, optimal squat performance requires maintaining a neutral head position and minimizing head movement during the movement. Consequently, the high MDC values may reflect both the intrinsic difficulty of standardizing cervical position and the technical challenge of digitally tracking this segment, highlighting the need to implement strategies to improve head stabilization during the squat.

Regarding the test–retest reliability of the protocol, ICC values ranged from 0.64 (moderate) to 0.98 (excellent). Notably, despite this variability, the vast majority of kinematic variables fell within the ‘good’ or ‘excellent’ reliability range, supporting the robustness of the protocol over time. These findings are consistent with previous Kinovea test–retest studies reporting ICCs > 0.85 [31,32].

In terms of test–retest reliability focusing on the primary kinematic variables, the Bland–Altman analysis (Figure 5) underscores the protocol’s temporal stability, which is essential for longitudinal follow-ups. While systematic biases remained exceptionally low across days, a predictable widening of the 95% limits of agreement (LoA) occurred compared to intra-rater findings. This incremental variance reflects natural day-to-day biological variability in movement execution and squat depth. This trend was most evident in hip flexion at P2 (95% LoA: −12.71° to 14.72°), where subtle out-of-plane compensatory movements are inevitably projected onto the 2D analysis plane. Nevertheless, visual inspection of the Bland–Altman plot showed a relatively balanced scatter of differences, with few apparent outliers. This pattern suggests a broadly stable error distribution and supports the potential robustness of the tracking protocol for sequential clinical assessments.

Our results are consistent with those reported by Soylu et al. [58], who, when comparing Kinovea with the D-Wall system in the overhead squat, obtained ICCs in the sagittal plane ranging from 0.79 to 0.99. In our study, knee flexion showed an almost identical ICC (0.98 vs. 0.99), but with a significantly lower SEM (1.98° vs. 6.06°). This greater absolute precision in our data suggests that the digitization protocol used allows for more accurate capture of joint position. However, for hip flexion, we obtained an ICC of 0.82 compared to 0.99 reported by Soylu et al. This discrepancy, despite a SEM of 5.15°, may be explained by differences in the squat depth achieved by the participants.

A critical finding was observed for variables such as head angle (MDC = 17.65–26.43°) and medial-lateral knee deviation at P2 (MDC = 39.61°). These values suggest limited clinical sensitivity for detecting subtle changes between sessions with 2D program. However, as noted previously, for variables such as head position, the clinical objective is not typically to increase range of motion but rather to achieve motor stabilization. Therefore, the utility of these measures lies more in monitoring whether the subject remains within neutral ranges than in detecting substantial angular changes.

To further scrutinize these high-MDC parameters, a dedicated Bland–Altman analysis was conducted to examine their absolute precision limits (Figure 6). Reflecting the cervical kinematics, head tracking exhibited the widest error margins both during the initial stance (P1 head angle: −1.82°; 95% LoA: −20.30° to 16.65°) and at maximum depth (P2 head angle: 5.03°; 95% LoA: −20.82° to 30.87°). Similarly, the frontal plane evaluation of the lower limb during maximum flexion revealed an expanded error distribution for the P2 medial–lateral knee deviation (3.68°; 95% LoA: −28.14° to 35.51°). Crucially, despite these wider confidence intervals, visual inspection of all three Bland–Altman plots did not reveal an evident heteroscedastic pattern, as the dispersion of the differences appeared relatively balanced across the observed measurement range. In addition, no clear pattern suggestive of systematic proportional bias was observed.

Together, these findings suggest that the 2D tracking protocol showed a reasonably consistent error distribution, even when capturing segments subject to high physiological variability or potential multiplanar movement artefacts. In the case of the knee in the frontal plane, the high MDC represents an inherent limitation of 2D photogrammetry, where rotational movements or displacements outside the sagittal plane are difficult to quantify with millimeters precision due to parallax error. In this regard, recent findings have questioned the robustness of 2D video analysis for clinical diagnostic applications, citing significant concerns regarding perspective error and marker-tracking accuracy [59].

In variables like head angle or knee in the frontal plane IMUs represent a more robust alternative [60,61,62], as they provide high-frequency, three-dimensional orientation data that is independent of camera position and field of view. Consequently, for clinical applications requiring the monitoring of rapid or multi-planar joint movements, inertial sensing systems offer a superior signal-to-noise ratio and greater sensitivity to change, effectively bypassing the limitations observed in the current 2D-based protocol [28].

The placement of anatomical markers is likely to be the most important factor influencing the accuracy and reliability of this study. Numerous studies have emphasized that consistent and precise marker alignment significantly affects measurement absolute precision and reliability [63]. This aspect was carefully controlled from the outset, as described in the methodology, since the markers were not removed from the participants’ skin between measurements, thereby ensuring consistent placement throughout.

Similarly, environmental factors, such as lighting conditions and the fixed positioning of the recording devices also influenced the accuracy.

Regarding clinical utility, Kinovea is highly appropriate for monitoring variables with low measurement error, such as foot pronosupination and horizontal pelvic alignment, where MDC values are notably low (0.99 cm–2.38°). The system also demonstrates acceptable reliability for sagittal-plane joint angles, such as knee flexion and pelvic tilt, with MDC values ranging from 5.48° to 7.87°. Conversely, clinicians should exercise caution when monitoring variables with high measurement error, such as head angle or frontal-plane knee dynamics, where MDC values can exceed 20°. For these parameters, subtle clinical changes are likely to be masked by the measurement error inherent in 2D video analysis; thus, for such variables, more precise 3D motion capture systems are recommended.

### Study Limitations

Regarding the sample characteristics, although the standardized marker placement minimized errors, accuracy remains inherently limited by soft tissue artifacts, particularly in participants with higher BMIs. Furthermore, the inclusion of a wide age range (20–71 years) and a modest sample size for multi-variable analysis introduced biomechanical heterogeneity; nevertheless, this diversity intentionally enhances the generalizability of our results, proving the protocol’s reproducibility across a heterogeneous, real-world clinical population. For this reason, the menstrual cycle phase was not recorded due to the large age variability spanning both pre- and post-menopausal participants. Furthermore, as this is a reliability study rather than an investigation into sex-specific biomechanical differences, any potential cycle phase effects are absorbed into the between-session variability, rather than confounding a between-group comparison.

In terms of technological constraints, the use of a 2D system for a 3D motion analysis represents an inherent limitation, as it cannot capture transverse plane rotations, potentially absorbing out-of-plane compensatory movements. In line with these optical limitations, markers located near the edges of the field of view—such as the head position—were subjected to minor parallax error and radial lens distortion. Consequently, future research could optimize accuracy either by focusing on a single target variable aligned with the camera’s optical axis or by employing advanced systems with automated parallax correction.

Finally, concerning movement execution, no strict standardization was enforced for squat tempo or depth, allowing participants to perform the exercise at a self-selected pace until maximum knee flexion. While this mirrors real-world physiotherapy assessments, it introduces subtle between-session variability, which, coupled with tracking anatomical landmarks on different days or the placement of markers on clothing over these landmarks, may account for the higher MDC values observed in specific postural variables.

## 5. Conclusions

The Kinovea 2D photogrammetry system demonstrates good to excellent intra-rater and moderate to excellent test–retest reliability for the kinematic assessment of the squat. Conversely, frontal-plane knee and head angles exhibit higher MDCs, marking an inherent limitation of 2D photogrammetry due to multi-planar movement and parallax. Consequently, while Kinovea is robust for sagittal clinical tracking, precise tracking of these specific parameters warrants three-dimensional or inertial sensing (IMU) alternatives.

## Figures and Tables

**Table 1:** Kinovea images of the angles measured for each variable.

Variable			Variable		
Head angle	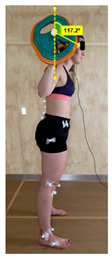	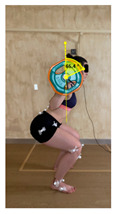	Knee flexion	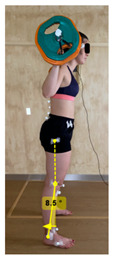	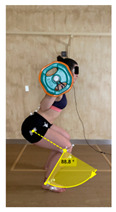
Lumbar lordosis	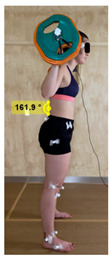	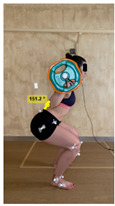	Ankle flexion	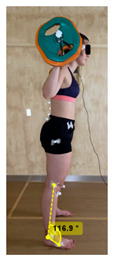	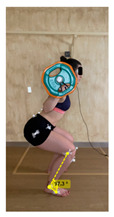
Horizontal pelvic alignment from a sagittal plane	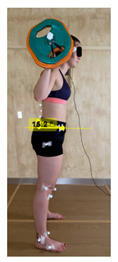	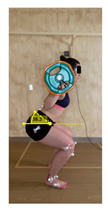	Medial-lateral knee deviation	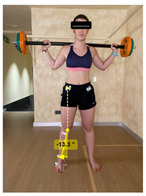	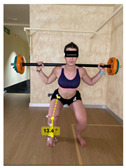
Trunk forward tilt	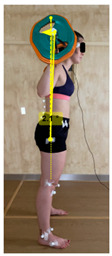	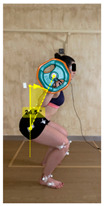	Horizontal alignment of the pelvis from a frontal plane	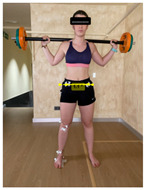	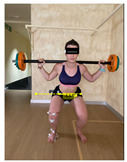
Hip flexion relative to the ASIS	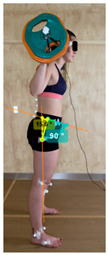	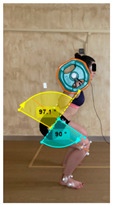	Foot pronosupination	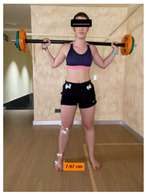	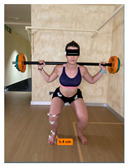

ASIS: Anterior superior iliac spine.

**Table 2:** Descriptive analysis of the movement variables recorded throughout the squat using the Kinovea 2D System and its intra-rater analysis.

Variable	Position	1st Measurement (±SD)	2nd Measurement (±SD)	ICC± (95% CI)	SEM (°, cm) (95% CI)	MDC (°, cm) (95% CI)
Ankle flexion (°)	P1	122.76° ± 5.90°	121.39° ± 5.80°	0.95 (0.88–0.98)	1.32° (0.83–2.03°)	3.66° (2.30–5.62°)
	P2	101.60° ± 9.04°	101° ± 8.85°	0.95 (0.87–0.98)	1.98° (1.27– 3.23°)	5.48° (3.52–8.95°)
Knee flexion (°)	P1	9.05° ± 4.98°	9.29° ± 4.68°	0.96 (0.90–0.99)	0.94° (0.48–1.53°)	2.60° (1.33–4.24°)
	P2	92.39° ± 13.98°	90.84° ± 13.42°	0.98 (0.95–0.99)	1.90° (1.37–3.06°)	5.26° (3.79–8.48°)
Hip flexion relative to ASIS (°)	P1	22.06° ± 6.33°	22.76° ± 6.85°	0.94 (0.85–0.98)	1.55° (0.93–2.55°)	4.29° (2.58–7.06°)
	P2	98.35° ± 12.15°	98.15° ± 12.70°	0.91 (0.79–0.97)	3.65° (2.15–5.69°)	10.11° (5.96–15.76°)
Horizontal pelvic alignment from a sagittal plane (°)	P1	19.83° ± 4.93°	19.71° ± 5.06°	0.95 (0.89–0.99)	1.10° (0.50–1.66°)	3.05° (1.39–4.60°)
	P2	44.67° ± 8.56°	45.1° ± 9.69°	0.82 (0.6–0.93)	3.63° (2.26–4.79)	10.06° (6.28–13.28)
Trunk forward tilt (°)	P1	−2.05° ± 4.89°	−2.19° ± 4.80°	0.99 (0.99–0.99)	0.49° (0.15–0.49°)	1.36° (0.42–1.36°)
	P2	35.66° ± 8.64°	34.54° ± 8.43°	0.97 (0.94–0.99)	1.46° (0.85–2.09°)	4.04° (2.35–5.79°)
Lumbar lordosis (°)	P1	158.22° ± 8.73°	158.42° ± 9.06°	0.94 (0.85–0.98)	2.14° (1.26–3.45°)	5.93° (3.49–9.56°)
	P2	166.69° ± 8.65°	165.65° ± 7.88°	0.89 (0.74–0.96)	2.61° (1.65–4.21°)	7.23° (4.57–11.66°)
Head angle (°)	P1	101.13° ± 14.25°	104.36° ± 15.09°	0.87 (0.70–0.94)	5.14° (3.59–8.04°)	14.24° (9.94–22.27°)
	P2	66.43° ± 21.32°	64.49° ± 24.16°	0.95 (0.86–0.98)	4.77° (3.02–7.98°)	13.21° (8.36–22.11°)
Medial-lateral knee deviation (°)	P1	−7.54° ± 4.44°	−7.2° ± 4.03°	0.97 (0.92–0.99)	0.70° (0.42–1.20°)	1.94° (1.16–3.32°)
	P2	41.76° ± 24.5°	42.66° ± 23.46°	0.99 (0.98–0.99)	2.35° (1.31–3.39°)	6.51° (3.63–9.39°)
Standing pronosupination movement (cm)	P1	4.01 cm ± 0.82 cm	3.91 cm ± 0.79 cm	0.96 (0.88–0.99)	0.16 cm (0.08 cm–0.28 cm)	0.44 cm (0.22 cm–0.78 cm)
	P2	3.52 cm ± 0.81 cm	3.53 cm ± 0.89 cm	0.91 (0.78–0.97)	0.24 cm (0.15 cm–0.40 cm)	0.66 cm (0.42 cm–1.11 cm)
Horizontal pelvic alignment from a frontal plane (°)	P1	2.58° ± 2.07°	2.37° ± 1.75°	0.94 (0.83–0.98)	0.43° (0.27–0.79°)	1.19° (0.75–2.19°)
	P2	1.38° ± 1.43°	2.01° ± 1.57°	0.75 (0.5–0.90)	0.71° (0.47–1.06°)	1.97° (1.30–2.94°)

P1: initial squat position; P2: final squat position 2; SD: standard deviation; ICC: intraclass correlation coefficient; CI: confidence interval; SEM: standard error of measurement; MDC: minimal detectable change. All ICC values were statistically significant (*p* < 0.05 or *p* < 0.001). SEM and MDC values were calculated using the SD of the 1st Measurement.

**Table 3:** Descriptive analysis of the movement variables recorded throughout the squat using the Kinovea^®^ 2D System and their test–retest analysis.

Variable	Position	1st Measurement (±SD)	2nd Measurement (±SD)	ICC ± (95% CI)	SEM (°, cm)	MDC (°, cm)
Ankle flexion (°)	P1	122.76° ± 5.90°	122.58° ± 5.80°	0.65 (0.30–0.85)	3.49° (2.28–4.94°)	9.67° (6.33–13.68°)
	P2	101.60° ± 9.04°	102.38° ± 10.43°	0.88 (0.70–0.94)	3.13° (2.38–5.33°)	8.67° (6.59–14.76°)
Knee flexion (°)	P1	9.05° ± 4.98°	9.06° ± 4.35°	0.72 (0.40–0.88)	2.64° (1.73–3.86°)	7.31° (4.78–10.69°)
	P2	92.39° ± 13.98°	92.28° ± 13.73°	0.98 (0.95–0.99)	1.98° (1.40–3.13°)	5.48° (3.87–8.66°)
Hip flexion relative to ASIS (°)	P1	22.06° ± 6.33°	21.53° ± 7.98°	0.86 (0.70–0.95)	2.37° (1.60–3.92°)	6.56° (4.43–10.86°)
	P2	98.35° ± 12.15°	99.34° ± 10.97°	0.82 (0.60–0.92)	5.15° (3.44–7.68°)	14.28° (9.52–21.30°)
Horizontal pelvic alignment from a sagittal plane (°)	P1	19.83° ± 4.93°	19.17° ± 4.65°	0.81 (0.59–0.92)	2.15° (1.39–3.16°)	5.96° (3.87–8.75°)
	P2	44.67° ± 8.56°	43.95° ± 7.82°	0.89 (0.74–0.96)	2.84° (1.71–4.36°)	7.87° (4.74–12.10°)
Trunk forward tilt (°)	P1	−2.05° ± 4.89°	−2.37° ± 4.38°	0.77 (0.50–0.91)	2.35° (1.47–3.46°)	6.51° (4.07–9.58°)
	P2	35.66° ± 8.64°	34.11° ± 9.19°	0.85 (0.65–0.94)	3.35° (2.18–5.27°)	9.28° (6.04–14.60°)
Lumbar lordosis (°)	P1	158.22° ± 8.73°	157.10° ± 9.58°	0.75 (0.49–0.90)	4.37° (2.89–6.54°)	12.10° (8.01–18.12°)
	P2	166.69° ± 8.65°	167.43° ± 9.70°	0.91 (0.78–0.96)	2.60° (1.84–4.30°)	7.20° (5.10–11.91°)
Head angle (°)	P1	101.13° ± 14.25°	102.95° ± 15.69°	0.80 (0.60–0.92)	6.37° (4.23–9.47°)	17.65° (11.72–26.23°)
	P2	66.43° ± 21.32°	61.41° ± 21.04°	0.80 (0.58–0.92)	9.53° (6.03–13.80°)	26.43° (16.71–38.25°)
Medial-lateral knee deviation (°)	P1	−7.54° ± 4.44°	−7.52° ± 3.97°	0.86 (0.68–0.95)	1.66° (0.99–2.51°)	4.61° (2.75–6.97°)
	P2	41.76° ± 24.50°	38.07° ± 14.23°	0.66 (0.30–0.85)	14.29° (9.49–20.50°)	39.61° (26.30–56.82°)
Standing pronosupination movement (cm)	P1	4.01 cm ± 0.82 cm	4.03 cm ± 0.67 cm	0.81 (0.60–0.92)	0.36 cm (0.23 cm–0.52 cm)	0.99 cm (0.64 cm–1.43 cm)
	P2	3.52 cm ± 0.81 cm	3.70 cm ± 0.86 cm	0.71 (0.4–0.88)	0.44 cm (0.29 cm–0.65 cm)	1.21 cm (0.80 cm–1.80 cm)
Horizontal pelvic alignment from a frontal plane (°)	P1	2.58° ± 2.07°	2.45° ± 2.26°	0.90 (0.76–0.96)	0.65° (0.43–1.06°)	1.81° (1.19–2.94°)
	P2	1.38° ± 1.43°	1.92° ± 1.56°	0.64 (0.25–0.84)	0.86° (0.60–1.29°)	2.38° (1.66–3.57°)

P1: initial squat position 1; P2: final squat position 2; SD: standard deviation; ICC: intraclass correlation coefficient; CI: confidence interval; SEM: standard error of measurement; MDC: minimal detectable change. SEM and MDC values were calculated using the SD of the 1st Measurement. All ICC values were statistically significant (*p* < 0.05 or *p* < 0.001).

## Data Availability

Data available on request due to restrictions (e.g., privacy, legal or ethical reasons).

## Abbreviations

P1	Position 1
P2	Position 2
ICC	Intraclass Correlation Coefficient
IC	Confidence interval
SEM	Standard error of measurement
MDC	Minimal Detectable Change
LoA	Limits of Agreement
BMI	Body Mass Index
PHA	Horizontal Pelvic Alignment
ASIS	Anterior Superior Iliac Spine
PSIS	Posterior Superior Iliac Spine
ND	Navicular Drop
SD	Standard Deviation
